# ML216 Alleviates Age-Related Cardiac Fibrosis by Suppressing TGF-β1 Signaling Pathway

**DOI:** 10.3390/ijms27083425

**Published:** 2026-04-10

**Authors:** Wenbin Liu, Feng Cui, Xiaodan Huang, Na Liang, Jun Li

**Affiliations:** State Key Laboratory of Common Mechanism Research for Major Diseases, Department of Biochemistry and Molecular Biology, Institute of Basic Medical Sciences & School of Basic Medicine, Chinese Academy of Medical Sciences & Peking Union Medical College, Beijing 100005, China; 18511402929@163.com (W.L.); cfisman@163.com (F.C.); huangxiaodan0610@163.com (X.H.); leona_liang@126.com (N.L.)

**Keywords:** cardiac aging, fibrosis, apoptosis, TGF-β1

## Abstract

Cardiac fibrosis is a hallmark of cardiac aging and a major contributor to development of heart failure. However, therapeutic strategies that specifically target cardiac fibrosis remain limited. In this study, we demonstrate that small-molecule compound ML216 exerts protective effects against aging-associated or β-adrenoceptor agonist isoproterenol-induced cardiac fibrosis in vitro or in vivo. Mechanistically, ML216 inhibits transforming growth factor-β1 (TGF-β1) signaling by reducing TGF-β1 protein levels, thereby attenuating Mothers against decapentaplegic homolog (SMAD) phosphorylation and downstream induction of connective tissue growth factor (CTGF). This leads to a marked suppression of fibrotic genes *Col1a1*, *Cnn2*, and *Acta2*, ultimately resulting in reduced fibrosis. Additionally, the inhibition of the TGF-β1 pathway alleviates cardiomyocytes apoptosis, which may further limit inflammatory responses and contributes to the overall attenuation of cardiac fibrosis. Collectively, these findings demonstrate that ML216 mitigates cardiac fibrosis through the inhibition of TGF-β1 pathway-mediated fibrotic signaling and apoptosis, highlighting its potential as a therapeutic candidate for the treatment of cardiac fibrosis.

## 1. Introduction

Cardiac fibrosis, characterized by the excessive deposition of extracellular matrix (ECM) proteins such as collagens, is a central pathological feature of adverse cardiac remodeling in response to diverse stressors, including pressure overload, myocardial infarction, and physiological aging [1,2,3,4]. Age-related cardiac fibrosis contributes substantially to the progressive decline in cardiac compliance and functional capacity observed in the elderly population [3,5]. Although fibrosis initially serves as a compensatory adaptive response to tissue injury, its uncontrolled progression leads to myocardial stiffening and impaired diastolic function, representing a major independent risk factor for heart failure and cardiac arrhythmias, and ultimately contributing to increased mortality [6,7]. To date, no FDA-approved drugs specifically target cardiac fibrosis [8,9]. Most clinically used agents exert anti-fibrotic effects indirectly and demonstrate limited efficacy in directly targeting fibrosis processes [10,11,12], including renin–angiotensin–aldosterone system (RAAS) inhibitors [13,14], β-blockers [15], and anti-fibrotic agents such as Pirfenidone and Nintedanib [16,17]. More recently, novel therapeutic strategies targeting key fibrosis pathways, including transforming growth factor-β1 (TGF-β1) [18,19] and tyrosine kinases, have been actively explored in clinical trials [20]. However, despite these efforts, effective therapies for cardiac fibrosis remain an unmet clinical need.

The molecular mechanisms underlying pathological cardiac fibrosis involve intricate and interconnected regulatory networks. TGF-β1, a key isoform of the TGF-β superfamily and a central pro-fibrotic cytokine, activates SMAD2/3 (Mothers against decapentaplegic homolog 2/3) cascade as well as TAK1/p38 pathways [21,22], leading to the upregulation of pro-fibrotic genes including collagens and connective tissue growth factor (CTGF) [23,24,25]. The PI3K/Akt pathway also contributes to cardiac fibrosis by regulating cell survival, apoptosis and gene transcription through downstream effectors such as mTOR and FoxO1/3 [26]. This pathway is believed to be chronically activated under sustained cellular stress during aging, thereby exacerbating age-related cardiac fibrosis [27,28]. In addition, angiotensin II, the principal effector of the renin–angiotensin–aldosterone system (RAAS), promotes fibroblast proliferation and collagen production, in part by upregulating TGF-β1 expression [29,30,31]. Collectively, among these interconnected signaling pathways, TGF-β1 signaling serves as a central regulatory hub and represents a promising therapeutic target for pathological cardiac fibrosis.

Adverse cardiac remodeling under stress conditions promotes not only cardiac fibrosis, but also cardiomyocyte hypertrophy and apoptosis [32,33,34]. Cardiomyocyte apoptosis in response to stimuli enables the removal of damaged cells without eliciting inflammation; however, excessive or sustained apoptosis disrupts myocardial structure and contractile function, ultimately contributing to heart failure [34,35]. Emerging evidence indicates that irradiation- and chemotherapy-induced cardiomyocyte apoptosis can drive fibrosis [36,37], implying that the inhibition of apoptosis may represent a potential cardioprotective strategy. Excessive cardiomyocyte apoptosis recruits macrophages and neutrophils, which secrete pro-inflammatory and pro-fibrotic cytokines, thereby activating fibroblasts and establishing an apoptosis-driven fibrotic cycle in which cell death promotes fibroblasts activation [38,39,40]. Notably, the TGF-β1 signaling pathway exerts dual pathogenic roles in adverse cardiac remodeling by directly inducing the differentiation of quiescent fibroblasts into collagen-secreting myofibroblasts [41,42,43], and by promoting stressed cardiomyocyte apoptosis through the regulation of pro-apoptotic proteins, including members of the Bcl-2 and caspase families [44]. Therefore, the inhibition of the TGF-β1 pathway represents a promising therapeutic strategy for pathological cardiac remodeling; however, effective agents for such purpose remain limited.

ML216 is a well-characterized small molecule, the full chemical name of which is 1-[4-Fluoro-3-(trifluoromethyl) phenyl]-3-(5-(pyridin-4-yl)-1,3,4-thiadiazol-2-yl) urea (PubChem CID: 49852229). It is a specific ATP-competitive non-covalent inhibitor (IC_50_ = 1.8 μM) of Bloom syndrome helicase (BLM) [45,46], which is a member of the RecQ helicase family that plays a pivotal role in DNA damage response and repair pathways [47,48,49]. Given the essential function of BLM in maintaining genomic stability through DNA repair and homologous recombination [50,51], ML216 was initially explored for oncological applications, aiming to induce synthetic lethality in a specific cancer subtypes or sensitize tumor cells to DNA-damaging agents by impairing their DNA repair capacity [47,52]. However, preclinical and clinical investigations have made limited progress in advancing ML216 for cancer therapy, largely due to insufficient selective cytotoxicity toward malignant cells [47]. These findings suggest that a re-evaluation of ML216’s cellular targets may be warranted. Our previous studies indicate that ML216 may modulate broader cellular stress responses by stabilizing BLM protein from degradation, suppressing DNA damage-induced senescence and senescence-associated secretory phenotype (SASP) factor expression, and alleviating fibrosis while improving pulmonary function in both aged mice and bleomycin-induced idiopathic pulmonary fibrosis (IPF) mouse models [49]. In addition, a recent study reported that ML216 exhibits antiviral activity against porcine delta coronavirus (PDCoV) in vitro by inhibiting viral replication by targeting host BLM helicase [53]. These findings support the potential repurposing of ML216 for senescence-related pathologies beyond oncology. Based on this rationale, we investigated whether ML216 can ameliorate age-related cardiac fibrosis using integrated in vivo and in vitro approaches.

In this study, we demonstrate that ML216 exerts cardioprotective effects against age-related cardiac fibrosis in mice. Aged mice treated with ML216 showed significantly reduced heart weight, decreased cardiac collagen content, and lower expressions of pro-fibrotic genes. Mechanistically, ML216 inhibits the TGF-β1 signaling pathway by reducing aging- or stress-induced TGF-β1 proteins, thereby suppressing downstream SMAD phosphorylation and CTGF expression. In addition, ML216 attenuated TGF-β1-mediated cardiomyocyte apoptosis. Collectively, these finding indicates that ML216 confers cardiac protection by mitigating cardiac fibrosis and apoptosis through the inhibition of the TGF-β1 pathway, highlighting its potential as a novel therapeutic strategy for pathological cardiac fibrosis.

## 2. Results

### 2.1. ML216 Reduces Heart Weights of Aged Mice

**Figure 1:**
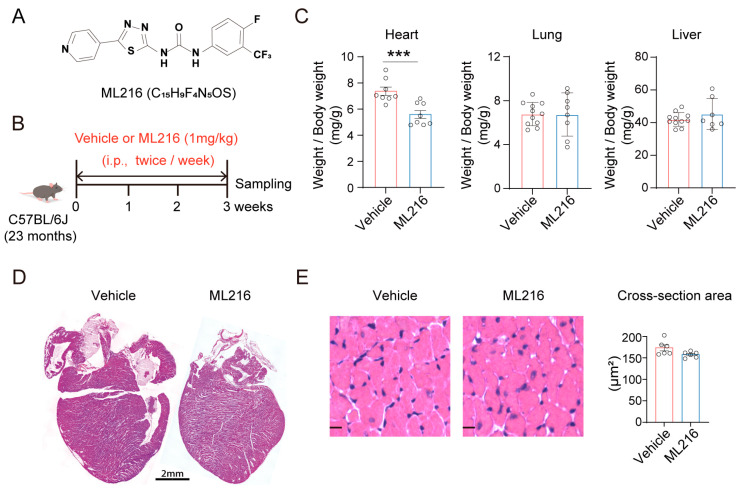
ML216 reduces heart weights in aged mice without affecting cardiomyocyte hypertrophy. (**A**) Chemical structure of ML216. (**B**) Schematic diagram of experimental design. The old mice (23-month-old female C57BL/6J) received intraperitoneal (i.p.) injections of vehicle or ML216 (1 mg/kg) twice weekly for 3 weeks (Vehicle: *n* = 11; ML216: *n* = 8). Samples were collected at the endpoint of the treatment. (**C**) Organ weights (normalized to body weight) for heart, liver, and lung; each dot represents a mouse. (**D**) Representative images of H&E-stained longitudinal sections of hearts from aged mice treated with ML216 or vehicle. Scale bar: 2 mm. (**E**) Quantification of cardiomyocyte cross-sectional area based on H&E-stained hearts sections of old mice treated with ML216 or vehicle. Scale bar, 10 μm, *n* = 6 mice for each group, 50 cells/mouse. Data are shown as mean ± SEM. *p* values were determined using two-tailed unpaired Student’s *t* test, *** *p* < 0.001 vs. vehicle.

Given that ML216 can reduce pulmonary fibrosis [49], we investigated whether ML216 exerts a similar effect in other organs. The available in vivo and prior evidence consistently indicates that ML216 at a concentration range of 0.5–1.5 mg/kg is well-tolerated in mice [49,52]. Accordingly, C57BL/6J mice (23 months) were intraperitoneally injected with ML216 (1 mg/kg) twice per week for three weeks. We found that ML216 significantly reduced heart weight but hardly affected the weights of the liver and lung (Figure 1A–C). To futher explore its effects at the tissue level, we further performed H&E staining of cardiac sections. Although the longitudinal section areas of ML216-treated mice appeared to be smaller than the control mice (Figure 1D), the cross-sectional area (CSA) of cardiomyocytes displayed no significant change enlargement (Figure 1E), suggesting it is unlikely that ML216 affects age-related cardiac hypertrophy.

### 2.2. ML216 Alleviates Age-Induced Cardiac Fibrosis in Mice

**Figure 2:**
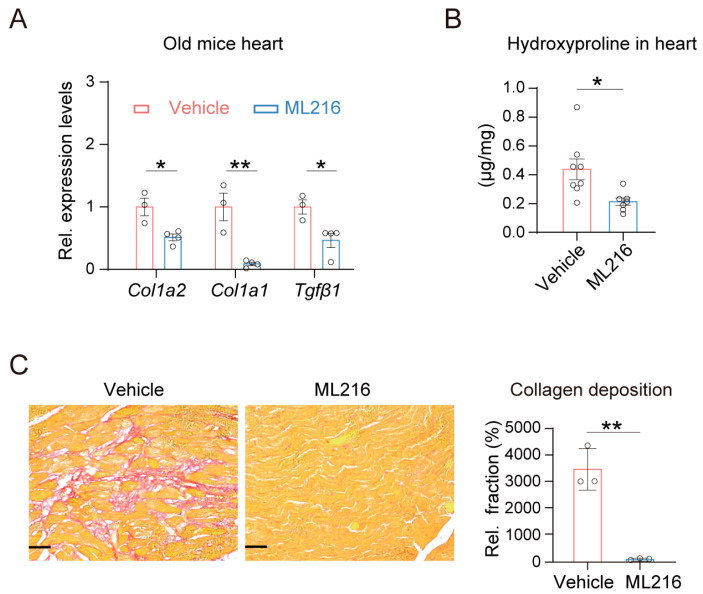
ML216 attenuates age-associated cardiac fibrosis in mice. (**A**) Relative expression of fibrotic genes *Col1a2*, *Col1a1*, and *Tgfβ1* in hearts of ML216-treated aged mice, examined by qPCR, n = 3–4 mice for each group. (**B**) Measurements of hydroxyproline contents (collagen quantification) in hearts of ML216-treated aged mice, *n* = 6–8 for each group. (**C**) Representative images of cardiac histological sections stained with picrosirius red staining. Scale bar: 50 μm. The quantification is shown on the right, *n* = 3 for each group. Data are expressed as mean ± SEM. *p* values were determined using two-tailed unpaired Student’s *t* test, * *p* < 0.05, ** *p* < 0.01 vs. vehicle.

Given that ML216 reduced the heart weights in aged mice without affecting cardiac hypertrophy, we next explored whether it influences cardiac fibrosis. Cardiac fibrosis, a pathological hallmark of the heart during aging, is the key factor that contributes to increased heart weight [54]. Our results showed that ML216 significantly downregulated the expression of collagen type I alpha 2 chain (*Col1a2*), collagen type I alpha 1 chain (*Col1a1*) and transforming growth factor-β1 (*Tgfβ1*), indicating a reduction in fibrotic signaling (Figure 2A). Consistent with this, measurement of the content of hydroxyproline, an essential component of collagen deposition in cardiac fibrosis [55], revealed a significant decrease in cardiac hydroxyproline content in the hearts of aged mice following ML216 treatment (Figure 2B). Picrosirius red-stained cardiac sections confirmed that ML216 attenuated collagen accumulation in the hearts of aged mice (Figure 2C). Together, these results indicated that ML216 effectively ameliorated cardiac fibrosis in aged mice.

### 2.3. ML216 Inhibits TGF-β1 Signaling Pathway

Although age-related cardiac fibrosis arises from complex processes, it shares key features and mechanisms with isoproterenol (ISO)-induced cardiac hypertrophy and fibrosis, including the induction of fibrosis-related genes including *Col1a1* (type I collagen) and *Ccn2* (CTGF), cardiomyocytes senescence, and the activation of pathological signaling pathways such as TGF-β1, NLRP3 inflammasome and oxidative stresses [56]. H9c2 cells, an immortalized myoblast cell line derived from embryonic rat heart that mimics immature cardiac muscle cells, is widely used to study cardiac hypertrophy, fibrosis and cardiotoxicity for their easy culture and ability to differentiate into cardiomyocytes under specific conditions. Thus, we used isoproterenol (ISO) to induce fibrosis in H9c2 cells as an in vitro system to study the mechanism underlying the anti-fibrotic effects of ML216.

**Figure 3:**
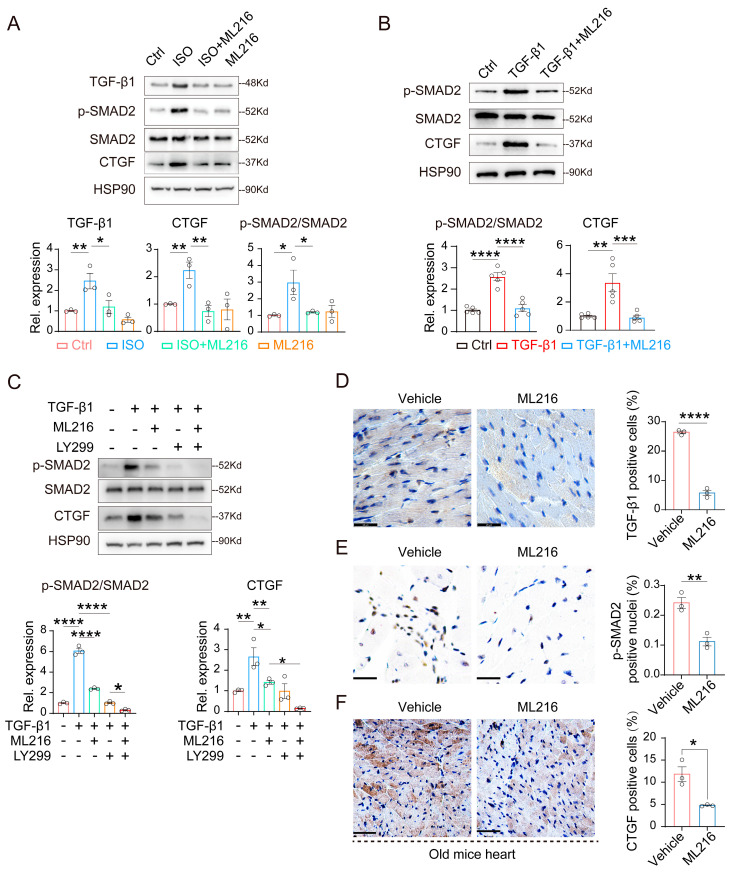
ML216 suppresses the TGF-β1 signaling pathway. (**A**) Western blot analysis and quantification of TGF-β1, p-SMAD2 (Ser255), SMAD2, and CTGF protein levels in H9c2 cells treated with isoproterenol (ISO, 40 μM), ML216 (20 μM), or both for 48 h, *n* = 3 biological replicates. (**B**) Western blot analysis and quantification of p-SMAD2 (Ser255), SMAD2, and CTGF protein levels in H9c2 cells treated with recombinant TGF-β1 proteins (5 ng/mL), or combined treatment of TGF-β1 (5 ng/mL) and ML216 (20 μM) for 48 h, *n* = 5 biological replicates. (**C**) Western blot analysis and quantification of p-SMAD2 (Ser255), SMAD2, and CTGF protein levels in H9c2 cells under the indicated treatment for 48 h. The concentrations for recombinant TGF-β1 protein, ML216, and the TGF-β receptor type inhibitor LY2157299 are 5 ng/mL, 20 μM, and 5 μM respectively, *n* = 3 biological replicates. (**D**–**F**) Representative images of immunohistochemical staining for TGF-β1, p-SMAD2 (Ser255), CTGF in cardiac sections of vehicle- or ML216-treated old mice. Scale bar: 25 μm (**D**,**E**); 50 μm (**F**). The quantification is shown on the right, *n* = 3 for each group. Data are presented as mean ± SEM. *p* values were determined using the one-way ANOVA (**A**–**C**) and two-tailed unpaired Student’s *t* test (**D**–**F**). * *p* < 0.05, ** *p* < 0.01, *** *p* < 0.001, **** *p* < 0.0001.

Transforming the growth factor-beta 1 (TGF-β1) signaling pathway is a master regulator of cardiac remodeling and fibrogenesis [22,57,58]. To determine a safe and effective concentration of ML216 for subsequent cellular experiments, we first performed a CCK-8 cytotoxicity assay. As shown in Figure S1, ML216 at concentrations up to 40 μM for 48 h did not cause significant cell death in H9c2 cardiomyocytes. Moreover, ML216 at 20 μM effectively suppressed the ISO-induced activation of the TGF-β1 pathway (Figure S2). Therefore, a concentration of 20 μM ML216 was used in all following cell-based assays. To explore the molecular mechanism of the anti-fibrotic effects of ML216, we conducted cellular experiments using isoproterenol (ISO) to stimulate fibrosis in H9c2 cardiomyocytes. Western blot results showed that ML216 significantly suppressed the ISO-induced elevation of TGF-β1 proteins. Consistently, ML216 repressed the downstream effector of TGF-β1, as evidenced by the decreased phosphorylation of SMAD2 at serine 255 and reduced expression of the key pro-fibrotic genes such as connective tissue growth factor (CTGF) (Figure 3A). Moreover, when fibrosis was induced by treatment with recombinant TGF-β1, ML216 similarly attenuated the phosphorylation of SMAD2 and the expression of CTGF at the protein level (Figure 3B), suggesting a direct effect on TGF-β1 signaling. In addition, co-treatment of ML216 with LY2157299, a well-characterized small-molecule inhibitor of TGF-β receptor type I (TβRI) [59,60], produced a synergistic effect on fibrosis repression, as evidence by further decreased phosphorylation of SMAD2 and CTGF levels compared to using ML216 alone (Figure 3C). To determine whether this mechanism also operates in vivo, we performed immunohistochemistry (IHC) analysis of cardiac tissue sections from aged mice. ML216 treatment significantly reduced the age-associated elevation of TGF-β1 (Figure 3D), as well as levels of phosphorylation of SMAD2 (p-SMAD2) and CTGF in hearts (Figure 3E,F). Collectively, these results demonstrate that ML216 mitigates cardiac fibrosis by inhibiting the TGF-β1/SMAD2/CTGF signaling pathway.

### 2.4. ML216 Alleviates TGF-β1-Mediated Fibrosis

**Figure 4:**
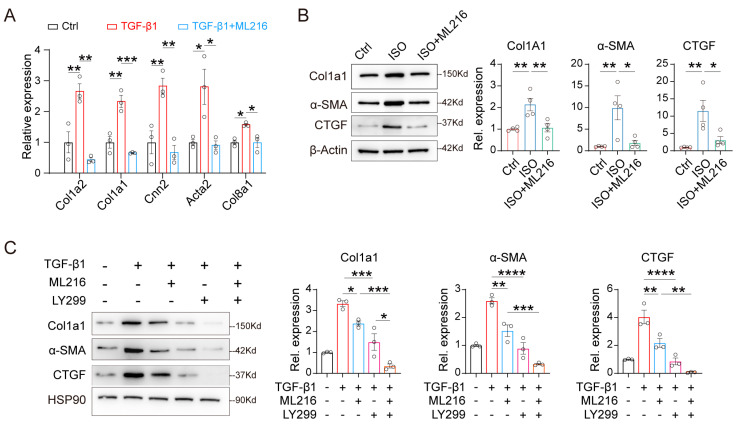
ML216 alleviates TGF-β1-mediated fibrosis. (**A**) Relative expression of *Col1a2*, *Col1a1*, *Cnn2*, *Acta2* and *Col8a1* in H9c2 cells treated with recombinant TGF-β1 proteins (5 ng/mL), or combined treatment of TGF-β1 (5 ng/mL) and ML216 (20 μM) for 48 h, test by qPCR, *n* = 3 biological replicates. (**B**) Western blot analysis and quantification of CTGF, α-SMA and collagen I protein levels in H9c2 cells treated isoproterenol (ISO, 40 μM), or combined treatment with ML216 (20 μM) for 48 h, *n* = 4 biological replicates. (**C**) Western blot analysis and quantification of CTGF, α-SMA and collagen I protein levels in H9c2 cells following the indicated treatments for 48 h. The concentrations for recombinant TGF-β1 protein, ML216, and LY2157299 are 5 ng/mL, 20 μM, and 5 μM respectively, *n* = 4 biological replicates. Data are expressed as mean ± SEM. *p* values were determined using the one-way ANOVA. * *p* < 0.05, ** *p* < 0.01, *** *p* < 0.001, **** *p* < 0.0001.

Given that ML216 inhibited the TGF-β1 pathway, we further investigated whether its anti-fibrotic effect is mediated through this signaling axis. Treating H9c2 cells with ML216 significantly suppressed the TGF-β1-induced upregulation of *Col1a2*, *Col1a1*, *Col8a1*, *Cnn2,* and *Acta2* (Figure 4A). Similarly, ML216 suppressed the ISO-induced upregulation of Col1A1, α-SMA (alpha-Smooth Muscle Actin) and CTGF protein levels (Figure 4B). Moreover, the co-treatment with ML216 and TGF-β1 receptor inhibitor LY2157299 produced a synergistic effect in repressing TGF-β1 protein-induced fibrosis in H9c2 cells (Figure 4C). These results indicate that the anti-fibrotic effect of ML216 are mediated, at least in part, via inhibition of the TGF-β1 signaling pathway.

### 2.5. ML216 Alleviates TGF-β1-Mediated Apoptosis In Vivo and In Vitro

**Figure 5:**
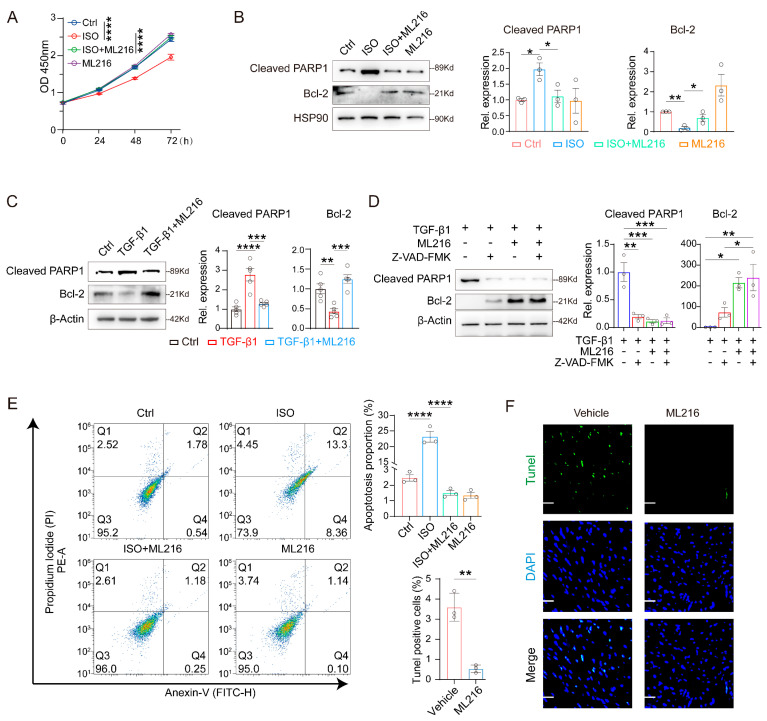
ML216 alleviates TGF-β1-mediated apoptosis in vivo and in vitro. (**A**) Survival curves of H9c2 cells treated with ISO (40 μM), ML216 (20 μM), or both for indicated time points, measured by CCK-8 assay, *n* = 3 biological replicates. (**B**) Western blot analysis and quantification of cleaved PARP1 and Bcl-2 protein levels in H9c2 cells treated with ISO (40 µM), ML216 (20 µM), or both for 48 h, *n* = 3 biological replicates. (**C**) Western blot analysis and quantification of cleaved PARP1 and Bcl-2 protein levels in H9c2 cells treated with recombinant TGF-β1 proteins (5 ng/mL), or combined treatment of TGF-β1 (5 ng/mL) and ML216 (20 μM) for 48 h, *n* = 5 biological replicates. (**D**) Western blotting analysis and quantification of cleaved PARP1 and Bcl-2 protein expression were performed in H9c2 cells following the indicated treatments for 48 h. The concentrations for ISO, ML216, Z-VAD-FMK are 40 μM, 20 μM, and 20 μM, respectively, *n* = 3 biological replicates. (**E**) Flow cytometry analysis of apoptosis of H9c2 cells treated with ISO (40 μM), ML216 (20 μM), or both for 48 h. H9c2 cells were stained with Annexin V-FITC and Propidium Iodide (PI), following the indicated treatment, *n* = 3 biological replicates. (**F**) Representative images of TUNEL staining in cardiac sections from vehicle- and ML216-treated aged mice. Scale bar: 25 μm. The quantification is shown on the right, *n* = 3 for each group. Data are shown as mean ± SEM. *p* values were determined using one-way ANOVA (**B**–**E**), two-tailed unpaired Student’s t test (**F**), and two-way ANOVA (**A**). * *p* < 0.05, ** *p* < 0.01, *** *p* < 0.001, **** *p* < 0.0001.

In addition to its role in fibrosis, the activation of the TGF-β1 pathway also often leads to apoptosis [61,62], a process elevated in aged or injured myocardium that further mediates fibrotic remodeling [63,64]. We therefore sought to explore whether ML216 could also reduce cardiomyocyte apoptosis in addition to its anti-fibrotic effect. The CCK-8 assay showed that ML216 improved the survival of H9c2 cells treated with ISO, while ML216 alone did not affect cell proliferation (Figure 5A). Western blot analysis revealed that ML216 significantly suppressed the ISO-induced upregulation of cleaved PARP1, a well-characterized marker of apoptosis [65,66], and restored the expression of Bcl-2, a key anti-apoptotic protein [67,68] (Figure 5B). Furthermore, ML216 reversed TGF-β1-induced increase in cleaved PARP1 and decrease in Bcl-2, similar to the effects of the pan-caspase inhibitor Z-VAD(OMe)-FMK [69,70] (Figure 5C, D). This result supported the fact that ML216 inhibits cardiomyocyte apoptosis, at least in part, through the suppression of TGF-β1 signaling. Flow cytometric analysis further confirmed that ML216 attenuated the ISO-induced apoptosis in H9c2 cells (Figure 5E). Consistent with in vitro data, ML216 treatment significantly reduced the number of TUNEL-positive apoptotic cells in the cardiac sections from aged mice (Figure 5F). Collectively, these data demonstrate that ML216 inhibits cardiomyocyte apoptosis by modulating the TGF-β1 pathway.

## 3. Discussion

This study provides the first evidence that ML216 attenuates age-related cardiac fibrosis and apoptosis through the inhibition of the TGF-β1 pathway. In addition to directly suppressing the TGF-β1 signaling cascade, ML216 may act synergistically with the TGF-β1 receptor inhibitor LY2157299, resulting in enhanced inhibition of TGF-β1 signaling. Given the central role of the TGF-β1 pathway in fibrosis across multiple organs [71,72,73,74], the anti-fibrotic effect of ML216 may extend beyond the heart and warrants further exploration in other organs. Consistent with this possibility, our previous study demonstrated that ML216 alleviates pulmonary fibrosis in IPF mice models [49]. Although liver weight remained unchanged in this study, whether ML216 exerts protective effects against hepatic fibrosis remains to be determined.

ML216 was originally developed as an inhibitor of the BLM helicase; however, our findings demonstrate its anti-fibrotic activity, necessitating further investigation for its cellular targets, particularly those involved in the inhibition of TGF-β1 signaling. It remains unclear whether this effect is dependent on BLM. Our in vivo data suggest that ML216 suppresses TGF-β1 expression at the transcriptional level, leading to reduced protein level. Given that BLM, as a DNA repair enzyme, has been implicated in epigenetic regulations [75,76], it is possible that ML216 modulates TGF-β1 gene transcription through BLM-mediated epigenetic mechanisms, thereby influencing downstream fibrotic processes. Meanwhile, the biological functions of ML216 remain incompletely understood. Thus, a BLM-independent mechanism cannot be excluded, including potential interactions with other, as yet unidentified, molecular targets. Comprehensive characterization of ML216’s binding partners and the signaling pathways it regulates will be essential for elucidating its anti-fibrotic mechanisms.

This study demonstrates that ML216 effectively attenuates age-related cardiac fibrosis, expanding its known biological functions and highlighting its potential as a promising therapeutic candidate for cardiac fibrosis. In addition to fibrosis, cardiac aging is often accompanied by hypertrophy. Notably, ML216-treated aged mice showed little change in cardiomyocyte size despite a downward trend, suggesting that the lighter heart weights is primarily attributable to reduced fibrosis rather than alterations in cardiac hypertrophy. This observation may reflect the distinct pathological context of cardiac aging. Although TGF-β1 is also a well-known inducer of cardiomyocyte hypertrophy, its effects in the aged heart likely involve complex interactions with additional regulatory pathways, differing from those observed in more defined models such as pressure overload models. In this study, female mice were used, and the inclusion of both sexes would be important for future studies to assess potential sex-specific effects. To further explore ML216’s therapeutic potential in cardiac fibrosis, the incorporation of functional cardiac assessments and validation in additional disease animal models, such as young mice with transverse aortic constriction (TAC) surgery-induced fibrosis, which is more clinically relevant, would be valuable.

Although fibrosis of heart, lung and liver shares common features of excess extracellular matrix (ECM) and collagen build-up, there are subtle differences due to the organ’s specific function and structure. For example, the large mass and exceptional regenerative capacity of liver render it relatively insensitive to mild, focal fibrotic changes. And liver weight is predominantly determined by hepatocyte density, vascular perfusion, lipid content, and metabolic load factors that overwhelmingly dominate total organ mass, rather than the ECM. Lung fibrosis, often referring to idiopathic (idiopathic pulmonary fibrosis, IPF), is caused by environmental toxins, smoking, or chronic inflammation. An important difference distinguishing the fibrosis of IPF to that of cardiac fibrosis is the nature and extent of inflammation. While both the innate and adaptive immune systems have roles in IPF, the inflammatory response is far less active than in other forms of fibrosis [77,78,79]. This explains why ML216, targeting mainly TGF-β1 signaling, showed no effect on lung fibrosis. In contrast, emerging evidence suggests that cardiac fibrosis mainly results from complications related to acute and prolonged inflammation [80,81], consistent with our finding that ML216 attenuated cardiac fibrosis via reducing TGF-β1 signaling.

ML216, first reported in 2013 [45], is a small-molecule inhibitor of the BLM helicase and is currently used primarily as a research tool rather than a clinically approved compound. Previous studies suggest that ML216 preferentially induces apoptosis in cancer cells with high BLM expression, while showing minimal toxicity to others [52], indicating a degree of selectivity. However, in vivo data remain limited. In support of its tolerability, our previous study using idiopathic pulmonary fibrosis (IPF) mice demonstrated that intraperitoneal administration of ML216 twice weekly for 5 weeks was well-tolerated [49]. Treated mice showed no significant changes in body weight and exhibited comparable serum levels of inflammatory markers, including IL-6, IL-8, and MCP-1, relative to control animals. In addition, ML216 treatment did not increase expression of the DNA damage marker p21 in liver or lung tissues, nor did it elevate fibrosis-associated readouts, including hydroxyproline content and the expression of fibrotic genes. Histopathological analysis further revealed no apparent tissue damage in the lung, with a reduction in senescence-associated β-galactosidase staining (SA-β-gal staining). While a comprehensive toxicological evaluation was not included in the present study, the dosage used here was within the reported well-tolerated dosage range (0.5–1.5 mg/kg) of ML216 in mice [49,52]. Further dedicated toxicological studies will be necessary to more fully define its safety profile, particularly in the context of future clinical translation.

## 4. Materials and Methods

### 4.1. Chemical Reagents and Antibodies

Isoproterenol hydrochloride (HY-B0468), ML216 (HY-12342) and TGF-β1 protein (HY-P7117) were purchased from MCE (Monmouth Junction, NJ, USA). Wheat Germ Agglutinin Conjugates (90-2504) was purchased from Btprobes (Shanghai, China). LY2157299 (T2510) and Z-VAD(OMe)-FMK (T6013) were purchased from Topscience (Shanghai, China). SBE-CD (H107) was purchased from Sigma (St. Louis, MO, USA). Environmentally Friendly Dewaxing Transparent Liquid (G1128), Sirius Red staining solution (G1018) and DAB substrate (G1212) were purchased from Servicebio (Wuhan, China). Antibodies were used at the following concentrations: anti-TGF-β1 antibody (SelleckChem, F1624, Houston, TX, USA, 1:1000,), anti-Phospho-Smad2 (Ser 255) antibody (SelleckChem, F1689, Houston, TX, USA, 1:1000), anti-Smad2 antibody (SelleckChem, F0309, Houston, TX, USA, 1:1000), anti-HSP90 alpha/beta antibody (Santa Cruz, sc-13119, Dallas, TX, USA, 1:1000), anti-GAPDH antibody (Servicebio, GB11002, Wuhan, China, 1:1000), anti-beta-Actin antibody (Servicebio, GB11001, Wuhan, China, 1:1000), anti-Cleaved PARP1 (Cell Signaling Technology, Asp214, Danvers, MA, USA, 1:1000), anti-BCL2 (Cell Signaling Technology, 2876, Danvers, MA, USA, 1:1000), HRP-conjugated anti-rabbit secondary antibody (Servicebio, GB23303, Wuhan, China).

### 4.2. Cell Culture

The H9c2 cell line was obtained from the Cell Resource Center (Institute of Basic Medical Sciences, CAMS/PUMC, Beijing, China). This cell line was maintained in DMEM (VivaCell, C3113, Shanghai, China) supplemented with 10% FBS, penicillin (100 U/mL), and streptomycin (100 µg/mL). The cells were maintained at 37 °C in a humidified incubator with an atmosphere containing 5% CO_2_ and were routinely subcultured using 0.25% trypsin EDTA (Servicebio, G4001, Wuhan, China) when they reached 70% confluence.

### 4.3. Mouse Treatment and Sampling

All C57BL/6J mice were purchased from SPF Biotechnology (Beijing, China) and maintained in specific pathogen-free (SPF) ventilated cages with ad libitum access to food and water. All animal procedures were approved by the Institutional Animal Care and Use Committee of Peking Union Medical College (ACUC-A02-2022-061). Aged female C57BL/6J mice (23 months old) were randomly allocated into two groups: Vehicle control group (*n* = 11) and ML216 treatment group (*n* = 8). ML216 was dissolved in sterile saline containing 10% DMSO and 20% SBE-β-CD administered at 1 mg/kg via intraperitoneal injection twice weekly for 3 weeks. Vehicle control mice received solvent alone. Body weight was monitored twice weekly. Upon completion of the 3-week treatment regimen, cardiac tissues were harvested for comprehensive analysis including gravimetric assessment of organ weights, histological evaluation via H&E staining to examine cardiac structure, and multi-modal fibrosis quantification through qPCR analysis of fibrotic genes, hydroxyproline-based collagen content measurement, and picrosirius red collagen visualization. Transversely sectioned left ventricles were used for all histology and immunohistochemistry experiments, except for Figure 1D, where hearts were sectioned along the longitudinal axis to evaluate valvular structures and overall chamber morphology. Concurrently, serum and tissue specimens were cryopreserved at −80 °C for subsequent molecular analyses.

### 4.4. Cell Viability Assay

H9c2 cells (1 × 10^3^ cells) were seeded into each well of 96-well plates, with a volume of 100 µL normal medium per well and subsequently incubated overnight. Next day, the cells were treated with ISO, ML216 and both for 24 h. Afterwards, 10 µL of CCK-8 solution (Vazyme, A311-02, Nanjing, China) was added, then incubation at 37 °C for 1–2 h. Soon afterwards, the optical density was measured at a wavelength of 450 nm utilizing a microplate reader (Synergy™ 4, BioTek Instruments, Inc., Winooski, VT, USA).

### 4.5. Apoptosis Assay

H9c2 cells (1 × 10^5^ cells) were seeded in 6-well plates and treated with ISO, ML216 and both for 24 h. Then, cells were harvested, washed with PBS twice, following which they were resuspended in binding buffer. According to the cell apoptosis detection kit’s instructions (Solarbio, CA1020, Beijing, China), cells were sequentially stained with Annexin V-FITC (Solarbio, Beijing, China) for 5 min in dark. Subsequently, the cells were incubated with Propidium Iodide (PI, Solarbio, Beijing, China) for 5 min at room temperature in dark before analysis by flow cytometry.

### 4.6. Western Blot

Following drug treatment, proteins from H9c2 cells were homogenized in lysis buffer (50 mM tris, pH 7.5, 150 mM NaCl, 0.5% NP-40) supplemented with protease inhibitor cocktail (MCE, HY-K0010, Monmouth Junction, NJ, USA), followed by SDS-PAGE and were electrotransferred to a 0.45 μm PVDF membrane (Millipore Corporation, IPVH00010, Billerica, MA, USA). Membranes were blocked with 5% non-fat dry milk in tris-buffered saline with Tween 20 (TBST) for 1 h and then incubated with primary antibodies overnight at 4 °C, followed by incubation with proper secondary antibodies. Blotting signals were visualized on Azure 200 Gel Imager (Azure Biosystems, Inc., Dublin, CA, USA). The band intensities were measured by ImageJ software (Version 8.0_345 64-bit).

### 4.7. Quantitative PCR

Total RNA was subsequently extracted using RNA isolator Total RNA Extraction Reagent (Vazyme, R401, Nanjing, China) per the manufacturer’s protocol. The cDNA was then synthesized using the HiScript™ III reverse transcription supermix (Vazyme, R333, Nanjing, China). Quantitative PCR was conducted with the Taq Pro Universal SYBR qPCR Master Mix (Vazyme, Q712-02, Nanjing, China). The sequences of the specific qPCR primers applied in this study are listed in Table 1.

### 4.8. Hydroxyproline Assay

Approximately 20 mg of heart tissue per mouse was collected from the left ventricles of the heart, minced, and subjected to acid hydrolysis (6 mol/L HCl, 200 μL) in a boiling water bath for 2 h. Following complete digestion, the samples were then neutralized with NaOH solution to reach pH to 6~8. Then the solutions were diluted to 400 μL, and centrifuged at 16,000 rpm (25 °C, for 20 min). The supernatants were collected for hydroxyproline colorimetric assay following the instruction using a commercial kit (Solarbio, BC0255, Beijing, China). The absorbance (560 nm) was measured by the SynergyH1 Multi-Mode Microplate Reader (Biotek, Winooski, VT, USA).

### 4.9. TGF-β1 Immunohistochemistry (IHC)

Paraffin-embedded heart sections (5 μm) were deparaffinized in xylene and dehydrated in pure ethanol (2 × 10 min) followed by gradient ethanol (95%, 90%, 80%, 70%; 5 min each). Antigen retrieval was performed in sodium citrate buffer (10 mM, pH 6.0) at 95 °C for 20 min. Endogenous peroxidase activity was quenched with 3% H_2_O_2_ for 15 min. Sections were blocked with 5% fetal bovine serum in PBS for 30 min and incubated with anti-TGF-β1 antibody (1:200) in 1% BSA/PBS at 4 °C overnight. After washing three times with PBST, sections were incubated with HRP-conjugated anti-rabbit secondary antibody (1:400) for 1 h at room temperature. Signals were developed using DAB substrate. Sections were counterstained with hematoxylin, dehydrated, cleared in xylene, and mounted with neutral balsam. Images were acquired under Leica DMi8 (Leica Microsystems GmbH, Wetzlar, Germany). TGF-β1-positive area was quantified using ImageJ by measuring optical density.

### 4.10. TUNEL Immunofluorescence (IF)

Heart sections were deparaffinized and rehydrated as above. After proteinase K digestion (20 μg/mL, 37 °C, 15 min), followed by PBS washes (3 × 5 min). Permeabilization employed 0.1% Triton X-100 at room temperature for 20 min, with subsequent PBS washes. After brief drying, tissues were equilibrated in equilibration buffer (10 min), then incubated with TUNEL reaction mix (TDT:dUTP:buffer = 2:5:50) in a humid chamber at 37 °C for 2 h. Then, sections underwent DAPI nuclear staining (10 min, dark), PBS washes, and mounting with anti-fade medium. Fluorescence microscopy (Leica DMi8, Wetzlar, Germany) examined images with DAPI (UV 358 nm; blue) and FITC (488 nm; green). TUNEL-positive nuclei were counted per sample and expressed as a percentage of total DAPI-stained nuclei.

### 4.11. Picrosirius Red Staining

The paraffin sections were immersed in sequence in Environmentally Friendly Dewaxing Transparent Liquid I for 20 min, Environmentally Friendly Dewaxing Transparent Liquid II for 20 min, Anhydrous ethanol I for 5 min, Anhydrous ethanol II for 5 min, 75% Ethyl alcohol for 5 min and then rinsed with tap water. Then, the slides were stained in Sirius Red staining solution for 8 min and then dehydrated quickly with two or three cylinders of anhydrous ethanol. The sections were put into the clean xylene for 5 min and sealed with neutral gum. Brightfield images were acquired under a light microscope (Evos FL Auto 2, Thermo Fisher Scientific Inc., Waltham, MA, USA). Collagen area percentage was quantified using ImageJ color thresholding.

### 4.12. Data Analysis

Experimental data are presented as mean ± SEM. Differences among multiple groups were evaluated using either ordinary one-way ANOVA followed by Tukey’s multiple comparisons test or two-way ANOVA followed by Sidak’s multiple comparisons test. All statistical analyses were conducted utilizing GraphPad Prism software Version 8.0 (GraphPad Inc., San Diego, CA, USA). Significance levels of statistics are indicated as * *p* < 0.05, ** *p* < 0.01, *** *p* < 0.001 and **** *p* < 0.0001.

## 5. Conclusions

Here we show that the small-molecule compound ML216 effectively attenuates age-related cardiac fibrosis in mice. This effect is likely mediated by reduced TGF-β1 protein level and subsequent inhibition of SMAD2/CTGF-associated fibrosis and apoptosis in cardiomyocytes. These findings not only expand the understanding of biological function of ML216 but, more importantly, identify ML216 as a promising candidate for treating cardiac fibrosis, an area of substantial unmet clinical need.

## Figures and Tables

**Table 1:** Specific primers for qPCR.

Gene Name	Species	Nucleotide Sequence (5′-3′)
*Ppia*	*mouse*	CATACAGGTCCTGGCATCTTGTC AGACCACATGCTTGCCATCCAG
*Hprt*	*mouse*	CTGGTGAAAAGGACCTCTCGAAG CCAGTTTCACTAATGACACAAACG
*β-Actin*	*mouse*	GTGACGTTGACATCCGTAAAGA GCCGGACTCATCGTACTCC
*Col1a2*	*mouse*	TTCTGTGGGTCCTGCTGGGAAA TTGTCACCTCGGATGCCTTGAG
*Tgfβ1*	*mouse*	TGATACGCCTGAGTGGCTGTCT CACAAGAGCAGTGAGCGCTGAA
*Col1a1*	*mouse*	GAAGGAACCTAACCATCTGGCA AGGAATAGAACGGTCTCTCCCA
*Hprt*	*rat*	AAATGGCGTTGGACTTGACATG ATGTAATCCAGCAGGTCAGCAA
*Col8a1*	*rat*	AAGGGCAGCTAACGGAAACATA TGGTGGTATCTGAGGAGGGATT
*Col1a1*	*rat*	AAGGCTCTAGAAAGAACCCTGC GGATGCAGGTTTCACCAGTAGA
*Col1a2*	*rat*	AAGGCTCTAGAAAGAACCCTGC GGATGCAGGTTTCACCAGTAGA
*Acta2*	*rat*	TAGAACACGGCATCATCACCAA CAGAGTCCAGCACAATACCAGT
*Cnn2*	*rat*	TCCATCCCCAAAATCAACCGAT CGTCAAAGTTTCGCTCCTGTTT

## Data Availability

The original contributions presented in this study are included in the article/Supplementary Materials. Further inquiries can be directed to the corresponding author.

## Supplementary Materials

The following supporting information can be downloaded at: https://www.mdpi.com/article/10.3390/ijms27083425/s1.

## Abbreviations

The following abbreviations are used in this manuscript:

a-SMA	alpha-Smooth Muscle Actin
BLM	Bloom syndrome helicase
CTGF	Connective tissue growth factor
ECM	Extracellular matrix
IPF	Idiopathic pulmonary fibrosis
ISO	Isoproterenol
ML216	1-[4-Fluoro-3-(trifluoromethyl) phenyl]-3-(5-(pyridin-4-yl)-1,3,4-thiadiazol-2-yl) urea
PI	Propidium Iodide
SMAD2	Mothers Against DPP Homolog 2
TAC	Transverse aortic constriction
TGF-β1	Transforming growth factor-β1
