# Supplementary material for: ML216 Alleviates Age-Related Cardiac Fibrosis by Suppressing TGF-β1 Signaling Pathway
Source: Int J Mol Sci. 2026 Apr 10;27(8):3425. doi: 10.3390/ijms27083425 (PMC13116860; doi:10.3390/ijms27083425)
Supplement: Supplementary file 1 [file ijms-27-03425-s001.zip › Supplementary Figures.pdf]

Supplementary Figures

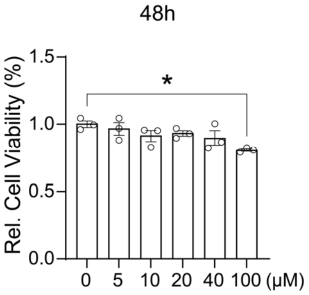

Supplementary Figure S1. Cytotoxicity of ML216 in H9c2 cells and selection of working concentration. CCK-8 assay was used to assess cell viability. H9c2 cells were treated with increasing concentrations of ML216 (0, 5, 10, 20, 40, 100 μM) for 48 h. n = 3 biological replicates. Data are presented as mean ± SEM. P values were determined using the one-way ANOVA. *p < 0.05.

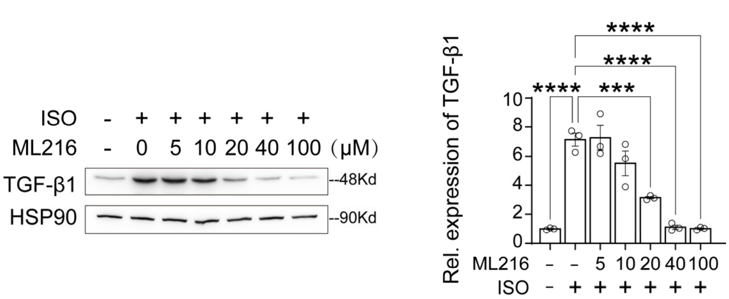

Supplementary Figure S2. Selection of ML216 concentration for suppressing ISO-induced TGF-β1 protein upregulation in H9c2 cells. Western blot analysis and quantification of TGF-β1 protein levels in H9c2 cells treated with ISO (40 μM) and increasing concentrations of ML216 (0, 5, 10, 20, 40, 100 μM)) for 48h, n = 3 biological replicates. Data are presented as mean ± SEM. P values were determined using the one-way ANOVA. ***p < 0.001, ****p < 0.0001.
